# Ankle Angle Prediction Using a Footwear Pressure Sensor and a Machine Learning Technique

**DOI:** 10.3390/s21113790

**Published:** 2021-05-30

**Authors:** Zachary Choffin, Nathan Jeong, Michael Callihan, Savannah Olmstead, Edward Sazonov, Sarah Thakral, Camilee Getchell, Vito Lombardi

**Affiliations:** 1 Department of Electrical and Computer Engineering, University of Alabama, Tuscaloosa, AL 35487, USA; zmchoffin@crimson.ua.edu (Z.C.); scolmstead@crimson.ua.edu (S.O.); esazonov@eng.ua.edu (E.S.); 2 College of Nursing, University of Alabama, Tuscaloosa, AL 35487, USA; mlcallihan@ua.edu (M.C.); smthakral@crimson.ua.edu (S.T.); cmgetchell@crimson.ua.edu (C.G.); valombardi@crimson.ua.edu (V.L.)

**Keywords:** ankle angle prediction resistive pressure sensor, machine learning, smart shoe

## Abstract

Ankle injuries may adversely increase the risk of injury to the joints of the lower extremity and can lead to various impairments in workplaces. The purpose of this study was to predict the ankle angles by developing a footwear pressure sensor and utilizing a machine learning technique. The footwear sensor was composed of six FSRs (force sensing resistors), a microcontroller and a Bluetooth LE chipset in a flexible substrate. Twenty-six subjects were tested in squat and stoop motions, which are common positions utilized when lifting objects from the floor and pose distinct risks to the lifter. The kNN (k-nearest neighbor) machine learning algorithm was used to create a representative model to predict the ankle angles. For the validation, a commercial IMU (inertial measurement unit) sensor system was used. The results showed that the proposed footwear pressure sensor could predict the ankle angles at more than 93% accuracy for squat and 87% accuracy for stoop motions. This study confirmed that the proposed plantar sensor system is a promising tool for the prediction of ankle angles and thus may be used to prevent potential injuries while lifting objects in workplaces.

## 1. Introduction

Every day, millions of workers report to their jobs with the hope of going home safely but many suffer musculoskeletal injuries and are unable to do so. Foot and ankle injuries accounted for 48,000 injuries with over 15,000 cases resulting in a visit to the emergency room in a 2019 study done by the U.S. Bureau of Labor Statistics [1]. Ankle injuries on average cause employees to take nine days off work for each injury occurrence [2]. Constant bending, twisting, walking and lifting creates an exertion of forces on the lower extremity. This is then transmitted from the bottom of the feet, through the talocrural joint (ankle), into the tibiofemoral joint (knee) and ultimately into the acetabulofemoral joint (hip) and the lumbar spine (lower back) [3]. These forces place stress on the joints and are amplified when the joint is in an abnormal position. This increased force and stress on the joints increases the risk of injury to the joints of the lower extremity.

Menz et al. [4] found that a pronation or an inward facing angle of the foot function among female subjects was associated with lower back pain. This discovery is further supported by the significant reduction in lower back pain recognized by participants who received an orthotic device to correct the excessive pronation of the foot in comparison with a placebo group [5]. The pronation of the foot directly correlates with the angle of the ankle, which directly influences the positioning of the knee. The positioning of the knee translates to alterations in the hip angle, applying pressure to the pelvis, which is transferred to the lumbar spine [3,5].

As workplaces and their conditions vary across the United States, the monitoring of the angles that employees position themselves in on the job is nearly impossible. Feedback related to the biomechanics of workers throughout the day would be invaluable to an employee, empowering them to recognize high-risk movements and take corrective action to minimize their risks. To date, real-time monitoring across diverse work groups is unavailable and, in most cases, not practical. Joint angle tracking has traditionally been completed using motion capture technology, which can be expensive and is not practical to use in the occupational setting. A current market solution is the Xsens full body motion capture system that uses IMUs to track the full movement of a person in combination with their analysis software and can cost over USD 7900. A solution was tested in [6] where the tracking of joint angles was based on FSRs as an alternative to the IMU. The FSRs allowed for an accurate reading of the applied force whilst being cost-effective compared with the current solutions.

In recent years, there have been many advances of technologies in detecting ankle angles. Camera systems were an early technology to detect angles; using human silhouettes, cameras could discern ankle-to-foot angles [7]. Given the system design, in daily activities this would be unfeasible. It was examined that a stationary position upon a force plate could accurately predict the ankle angle using the center of pressure of the human body [8]. In a sitting position, six wire displacement sensors were used to measure the ankle joint in the six degrees of freedom with a root mean square error of less than 2 [9]. Multiple IMUs have proven useful [10,11,12,13]; however, they are not feasible due to the complex setup for daily use, mechanical instability in installation and high cost. MRI (magnetic resonance imaging) [14], CT (computer tomography), ultrasound sonography [15] and X-rays [16] have been used to generate medical images to measure ankle angles but support only a single image at a stationary position. A SVR (support vector regression) machine learning technique was utilized to a high precision with a pre-recorded data analysis to find hip and knee joint motion data, which were then inserted into a predictive model [17]. A combined method of using X-rays and a deep learning technique was proposed to identify hip, knee and ankle angles on the unilateral lower limb [18]. However, little has been studied for the continuous and real-time prediction of ankle angles with a foot-based pressure sensor and a machine learning method. Machine learning is an emerging technique used in biomechanics. The prediction of falls using a multivariable logistic regression [19] was explored using observed gait patterns of stroke patients. Center of pressure foot insoles and IMUs used in a linear regression algorithm predicted the force upon the joint with high accuracy [20]. Fabric-based strain sensors in combination with a linear regression algorithm had a high accuracy when compared with an angle extracted from a high-speed camera [21]. Additional strain sensor work using a Fourier series analysis provided a root mean square error of less than 2 [22]. Electrode-based sensors placed on the proximal nerve trunk with the assistance of a recurrent neural network successfully predicted the ankle angle [23]. Electromyography sensors attached above the angle using a random forest algorithm had a great accuracy when compared against sample data of known angles [24]. However, no solutions provided a simple and compact sensor system that could detect angles based on the foot pressure.

In this paper, a footwear system is designed with the six FSRs and a microcontroller to measure pressure at the primary contact points of the foot. A kNN machine learning technique is applied to predict the angle of the joint. The predicted ankle angles are compared with the reference IMU data for validation. At the beginning of the study, we hypothesize that the developed footwear sensor system will be able to predict the ankle angle with a greater than 90% accuracy.

## 2. Materials and Methods

### 2.1. Insole Pressure Sensor System

A body in motion has force exerted on it from the ground known as a ground reaction force. This force varies depending upon the motion of the person and is distributed through the foot, into the ankle and up through the rest of the body. The positioning of the foot as it contacts the ground influences the pressure as it is distributed up and through the rest of the body. For this study, six FSR sensors were placed at the common pressure points to effectively capture the forces experienced by the foot due to changes in movement. When an FSR experiences mechanical stress, the resistivity changes and causes it to increase or decrease the currents flowing through it. The variation in resistance is used to estimate the amount of force applied to individual sensors. As the force increases, the resistance is usually represented by a range of resistances from 10 MΩ to 30 KΩ. The resistance curve is logarithmic with a linear increase in force [25]. As the force approaches the maximum rating of the sensor, the resistance changes become smaller. Given the known surface area of a single FSR, the pressure can be derived from the force.

**Figure 1:**
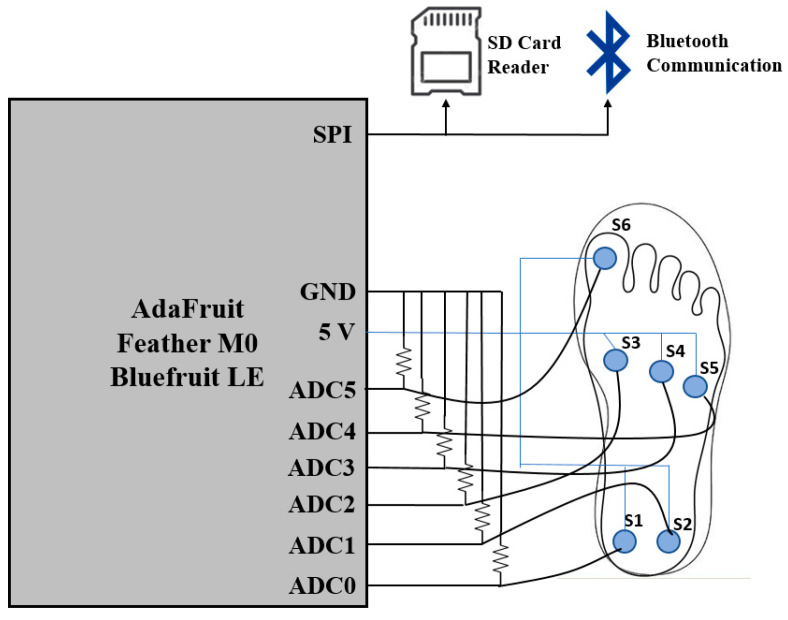
Schematic of the developed pressure sensor system.

The FSR sensors (FlexiForce A301) were integrated with a commercially available microcontroller fitted with a Bluetooth Low Energy module (Adafruit Feather M0 Bluefruit LE [26]), an SD card reader for data recording and a 3.7 V lithium-ion battery. The schematic of the pressure sensor system is depicted in Figure 1. The microcontroller was implemented with an ATSAMD21G18 ARM Cortex M0 processor with a clock frequency of 48 MHz and 3.3 V logic. The chip had 356 K of Flash memory and 32 K of RAM. The chip was programmed to convert the measured analog voltage to digital values via a USB-to-serial connection. The Bluetooth LE was realized with an nRF51822 chipset from Nordic. A computer code was written to communicate to the Bluetooth LE module via an UART connection profile and to save the pressure sensor data directly to a data collection unit, a smartphone (iPhone XS). An ADC (analog-to-digital converter) was connected to each FSR to detect the analogue signal in the pressure sensors. The drop-down resistors were connected to the ADC terminal to the ground pin and a threshold of voltage was set to be read. To provide flexibility and the solid connection of FSRs on the surface of the insole, etched thin copper strips were fabricated and glued on a bendable transparent substrate (cellulose acetate). The copper strips were routed to a common power line and six signal ADC terminals. An additional layer of cellulose acetate was placed on top to protect the strips. The data from the insole pressure sensors were recorded at a sampling frequency of 50 Hz, stored in the SD card in real-time and post-processed to train a machine learning algorithm to predict the ankle angles.

**Figure 2:**
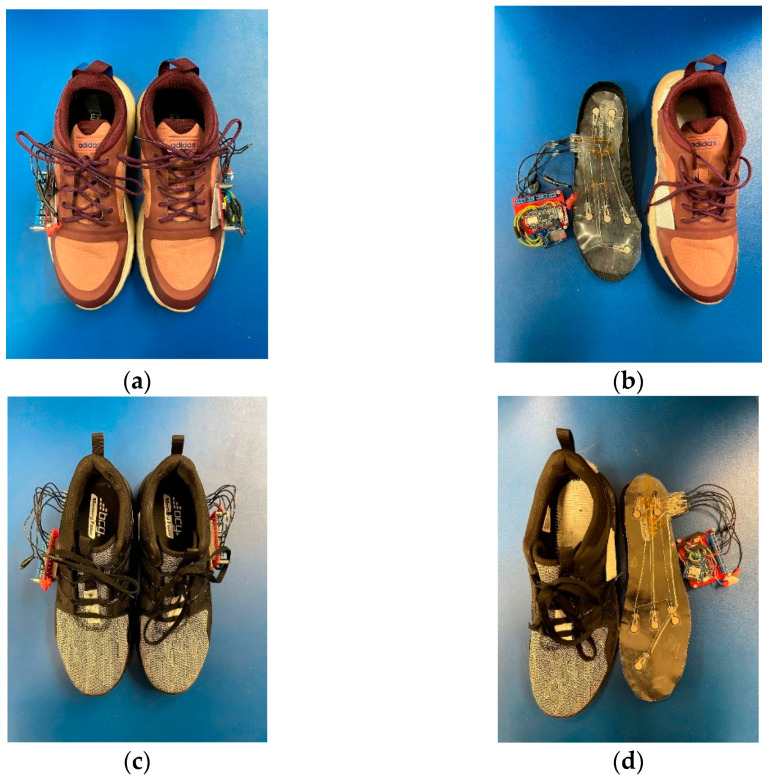
Ankle angle detection system: (**a**) women’s size 8.5 shoes with an integrated microcontroller and data transmission circuit; (**b**) insole with FSRs and a detection circuit for the women’s shoe; (**c**) man’s size 10.5 shoes with an integrated microcontroller and data transmission circuit; (**d**) insole with FSRs and a detection circuit for the man’s shoe.

Two different sized shoe insoles were developed to increase the pool of available participants. The two shoe sizes used were based{Mickle, 2010 #37} [27] which showed that in the United States the average male shoe size was 10.5 and the average female shoe size was 8.5. The developed sensor-integrated insoles and detection circuits for a woman and a man are displayed in Figure 2. The insole sensor was placed under the factory shoe insole. The thin and flexible strips were fed through a slit in the side of the shoe and attached to the detection and data storage circuit.

### 2.2. Motion Capture for Reference Data

**Figure 3:**
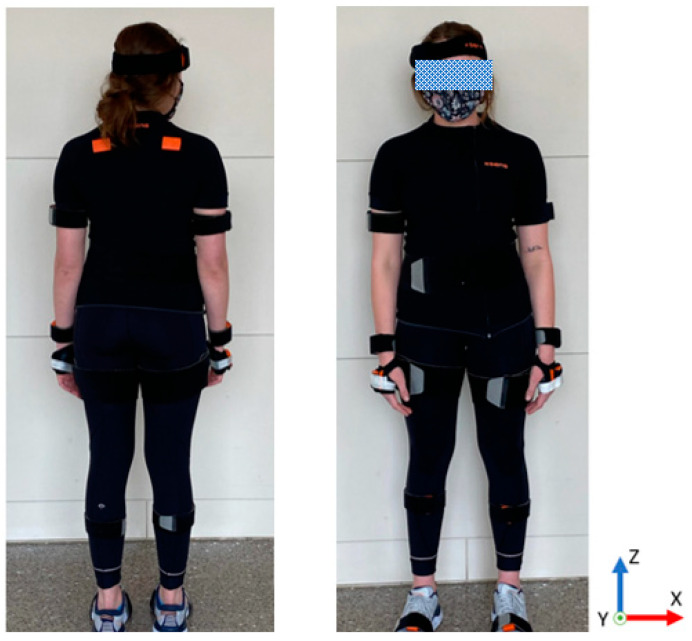
Full Xsens body suit used during testing.

Motion capture technology is the gold standard for the collection of kinematic data [28] and was used for this study to correlate and validate the angle of the ankle to the pressure across the feet. A commercially proven motion capture system, Xsens, utilized seventeen inertial measurement units (IMUs) placed on the body to identify the body segment orientation and the angle of the joints, as shown in Figure 3. Upon placement of the IMU devices for the study, the sensors were calibrated with the participant standing in the N-pose (upright position, head forward, arms resting at sides), walking forward and returning as per the manufacturer’s recommendations. Following the collection of the movements, the data were processed in the Xsens motion capture software and then through Visual 3D with the joint angles calculated based on the orientation of the distal body segment to the proximal body segment. For the ankle, this was the orientation of the foot relative to the shank.

### 2.3. Data Collection

#### 2.3.1. Sensor Synchronization

The synchronization between the proposed footwear sensor system and Xsens IMU systems was accomplished by having the participant raise their heels at the start and end of each movement. In the footwear insole system, this synchronizing motion generated a large pulse in the heel sensor signals providing a starting and ending point of the test. In the IMU devices, this produced a directional acceleration in the *x*-axis used to signal the start and finish of the movement allowing for the synchronization of the systems. Once the movement was finished with a known starting and ending point on both systems, both systems needed to be time scaled together. The Xsens IMU unit was configured to sample at 60 Hz then normalized to 1000 data points for the movement. The Xsens ankle angle data was then down-sampled to match with the footwear sensor data.

#### 2.3.2. Data Collection

The data collected from the footwear sensor system were stored in a text file format located on the SD card. The data contained a relative pressure reading from each FSR, which was calculated based on the force applied to the sensor that was then normalized and the time referenced to the startup time of the microcontroller. The time between each capture took roughly 20 ms ± 2 ms based on a 50 Hz sampling output rate. The ADC on the Feather M0 had a resolution of 10 bits, meaning it could detect the small changes of pressure that occurred when a greater force was applied.

**Figure 4:**
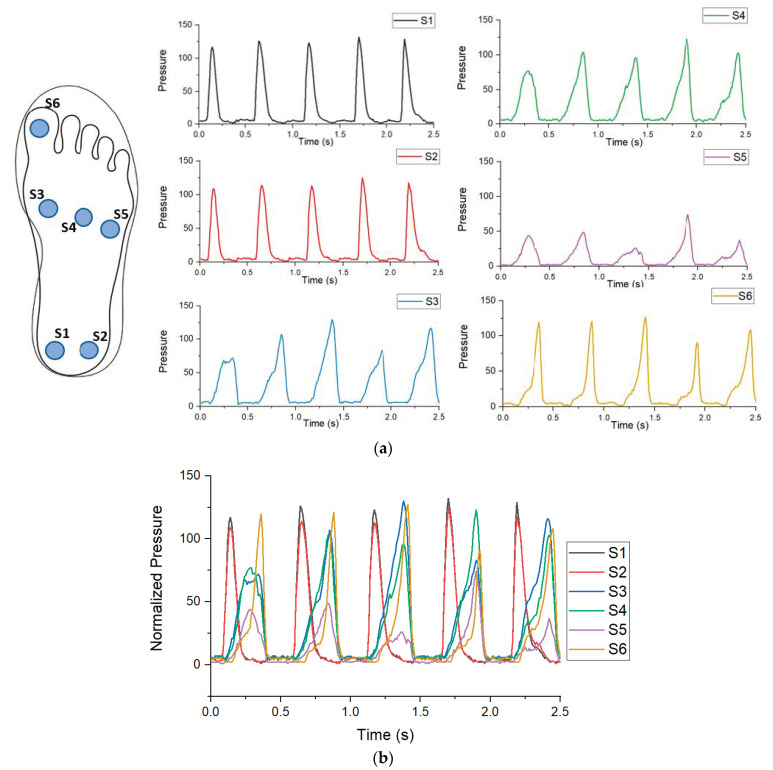
Right foot sensor output for walking: (**a**) pressure outputs of individual sensors; (**b**) combined sensor graph with a complete output of the insole.

Upon starting a test, the microcontroller recorded the relative pressure, which was the relative pressure at each sensor from 100 to 0 for each sensor. Data were then smoothed using a lowpass filter. S1 and S2 were located at the calcaneus while S3, S4 and S5 were located along the metatarsals. S6 was placed on the hallux. Demonstrated in Figure 4 is an example of five steps of a walking movement. Figure 4a contains the plots of the pressure recorded by each individual sensor. Images S1 and S2 display the heel striking of a foot starting a walking cycle. Images S3, S4 and S5 display a slow build of pressure as the participant walks forward. Image S6 slowly builds to a spike. Figure 4b displays all of the pressures combined from Figure 4a; together, this forms a pressure distribution over time showing greater pressure in one area over the other.

### 2.4. Experimental Procedure

For this study, a total of 26 participants were recruited and tested. The current study was authorized by the Institutional Review Board (IRB NO. 20-02-3356-A) at The University of Alabama. Of the 26 subjects, 11 males and 15 females participated with 12 subjects using size 10.5 shoes and 14 subjects using size 8.5 shoes. The participants had an average age of 22.9 years old and were recruited between October 2020 and May 2021. The participants were selected primarily based on shoe size to match the developed sensor. The participant information is featured in Table 1 below.

The experiments took place at The University of Alabama Capstone College of Nursing. Each participant was asked to complete a series of movements while wearing the pressure sensing shoes and Xsens IMU system. The participants were adequately educated of all motions before taking the action. The experiment was conducted after receiving informed written consent from all participants in the study. While participants completed the experiment, the insole system recorded the pressures and the Xsens system recorded the total body movement.

### 2.5. Movement Description

#### 2.5.1. Movement Rationale

The movements for the study were chosen from the approved movements by The University of Alabama IRB. The criterion for the evaluation of the movement was for the foot to be on the ground during the period of the test. Different movements will cause different areas to increase based on the foot placement and body angles. In the stoop and squat movement, there are definite changes in pressure that can be associated with the different angles of the ankle. The squat movement was chosen as it can be accurately controlled and is repeatable with the feet never leaving the ground. The stoop motion was chosen with the plant foot for both a realistic movement and a controlled repeatable movement.

#### 2.5.2. Squat

**Figure 5:**
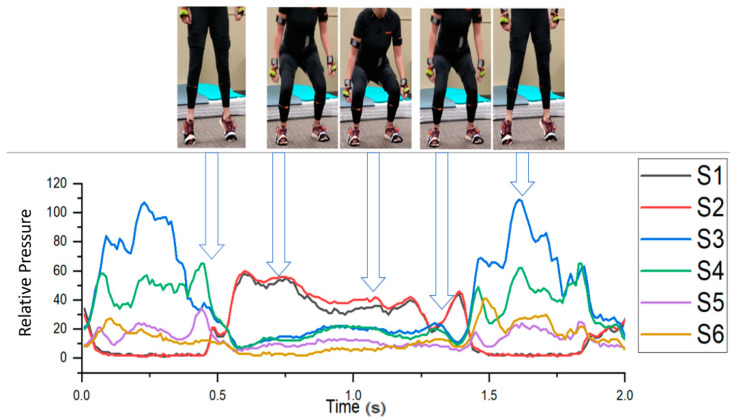
The squat motion with the accompanying sensor readout.

A squat, as defined in this study, started with the subject raising their heels off the ground to indicate the start of the motion. The subjects then entered a shallow squat position where the legs became parallel to the ground. The subjects then rose to a standing position and finished by raising their heels again to show the end of the motion. This movement was repeated a total of ten times per participant. The movement progression is illustrated below in Figure 5 with the accompanying sensor readout. As a participant progressed through the movement, S1 and S2 registered near zero then the sensor spiked to signal the start of the movement. As a squat progressed, a greater pressure was applied to the front of the foot, S3–S6. The squat then ended with S1 and S2 dropping to near zero, which signaled the end of the movement.

#### 2.5.3. Stoop

**Figure 6:**
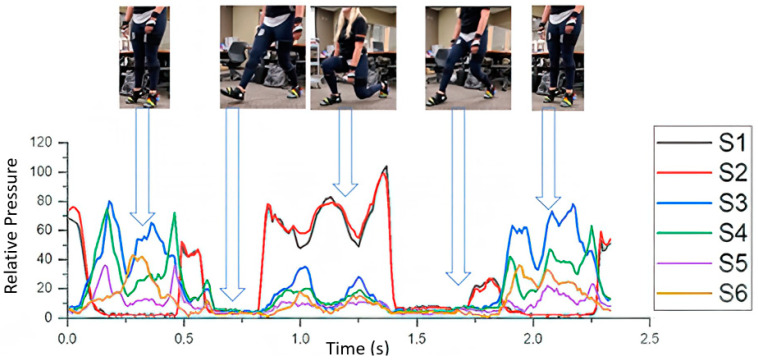
The stoop motion of the experiment with the accompanying sensor readout for the left foot of the movement.

A stoop motion was the next motion selected; this movement was chosen to test various angles while applying different pressures to the sensors. The stoop commenced with the subject raising their heels off the ground. The participant then lunged forward on the instructed side, completing a 90 degree bend of the knee. The subject then pushed off the ground and returned to a standing position. Each participant then finished with a heel raise to signify the end of the test. The movement was done ten times per leg, totaling 20 times per participant. The movement progression is illustrated below in Figure 6 with the accompanying sensor readout. At the beginning of the movement, a large spike signaled the start of the test with a heel strike to the ground on S1 and S2. As the movement continued, the left foot came off the ground and the sensor readings fell to zero. The foot returned to contact with the ground from the heel, shown with S1 and S2 rising, with the S3, S4 and S5 rising as the center of pressure of the foot moved towards the front of the foot. The foot then pushed off the ground with a final rise in S1 and S2 before going to zero as the foot was off the ground. The movement then ended with a heel raise.

### 2.6. Machine Learning to Predict Ankle Angles

**Figure 7:**
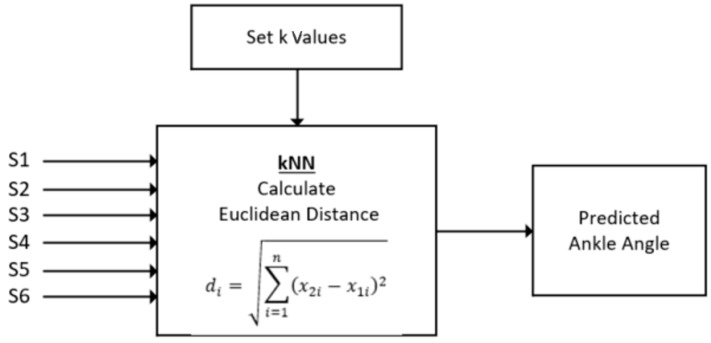
Block diagram of the kNN classification applied to this study.

A nonparametric density classifier, a kNN, was used to predict the ankle angles. Figure 7 shows the flow diagram of the kNN whose input was the six sensor pressure data and output were the ankle angle predicted. All participant data were combined together and split into two datasets; 80% for training the surrogate model and 20% of the other remaining dataset for validation. The kNN was chosen using the MATLAB classification solver, which tests a skew of the classification-based algorithms and determines which one works based on the given dataset. In the design of the prototype, the kNN algorithms were computationally efficient and allowed for a rapid design. The model calculated the Euclidean distance between the points in the training dataset and the input points. Given a trained dataset and the distance, the kNN searched and found the k-closest points in the input dataset to the trained dataset. The decision for the classification of the input points was made based on the sum of the distances and the class provided that the closest distance was selected as the value predicted. The number of neighbors remained at 10 and the optimal value was determined where the highest accuracy was achieved. In our dataset, a 6-input data sheet containing all sensors was inserted in the kNN algorithm and the optimal 10 values were chosen using MATLAB. A classification-based model was chosen compared with a linear regression-based model because the angle data were reported with an integer level accuracy.

## 3. Results

### 3.1. Machine Learning Result

**Figure 8:**
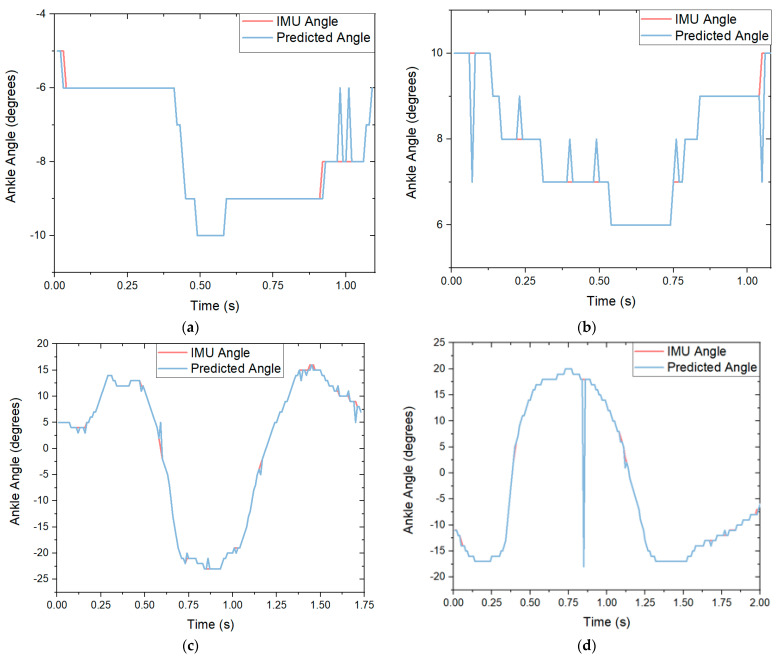
The Xsens-predicted angle compared with the algorithm-predicted ankle angle in the *y*-axis for each movement: (**a**) graph of a single squat movement on the left foot with an accuracy of 94.2%; (**b**) graph of a single squat movement on the right foot with an accuracy of 92.4%; (**c**) graph of a single right stoop movement on the left foot with an accuracy of 90.2%. (**d**) graph of a single left stoop movement on the right foot with an accuracy of 88.9%.

To predict the ankle angles, MATLAB R2019b was used. Various machine learning models including decision trees, neural networks and a discriminant analysis were considered to find an appropriate machine learning algorithm. It was found that a kNN algorithm [29] could predict the discrete ankle angles with the highest accuracy. Figure 8 shows the graphs of how the algorithm predicted the angle of a participant’s ankle in the Y direction over the duration of the movement. Figure 8a displays how the algorithm predicted the ankle angle compared with the Xsens IMU for a single squat for the left foot. The generated algorithm had a prediction accuracy of 93.6%. The accuracy for this movement performed better than trained by over 5%. Figure 8b shows the predicted accuracy of a single movement of a squat for the right foot. The movement was classified with an accuracy of 93.8%. Figure 8c displays the predicted angle versus the actual angle of the ankle of a right stoop movement on the left foot. The algorithm predicted the angle with an 89.5% accuracy. Figure 8d displays the predicted angle versus the actual angle of the ankle of a left stoop on the right foot. The algorithm predicted the angle with an 87.4% accuracy.

### 3.2. Predicting the Angle of a Movement

These tests used both feet to predict movements and used all participants. A standard holdout percentage of 20% of the data was used across all tests to validate the model. The squat movement was regarded for both feet. In the stoop movements, due to the fact that during the test one foot was off the ground causing a floating known value, the plant foot of the movement was regarded as it remained on the ground. The angle ranges in the Table 2 would not be the same. Given the Xsens unit tracking angles, the positive y angle would go to the right lateral of the body. The negative y angle would go to the left lateral of the body.

**Figure 9:**
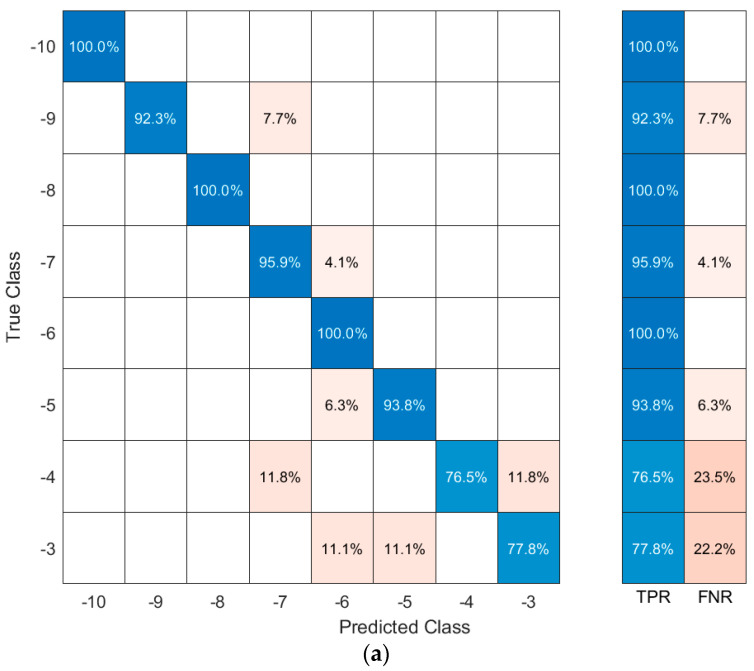

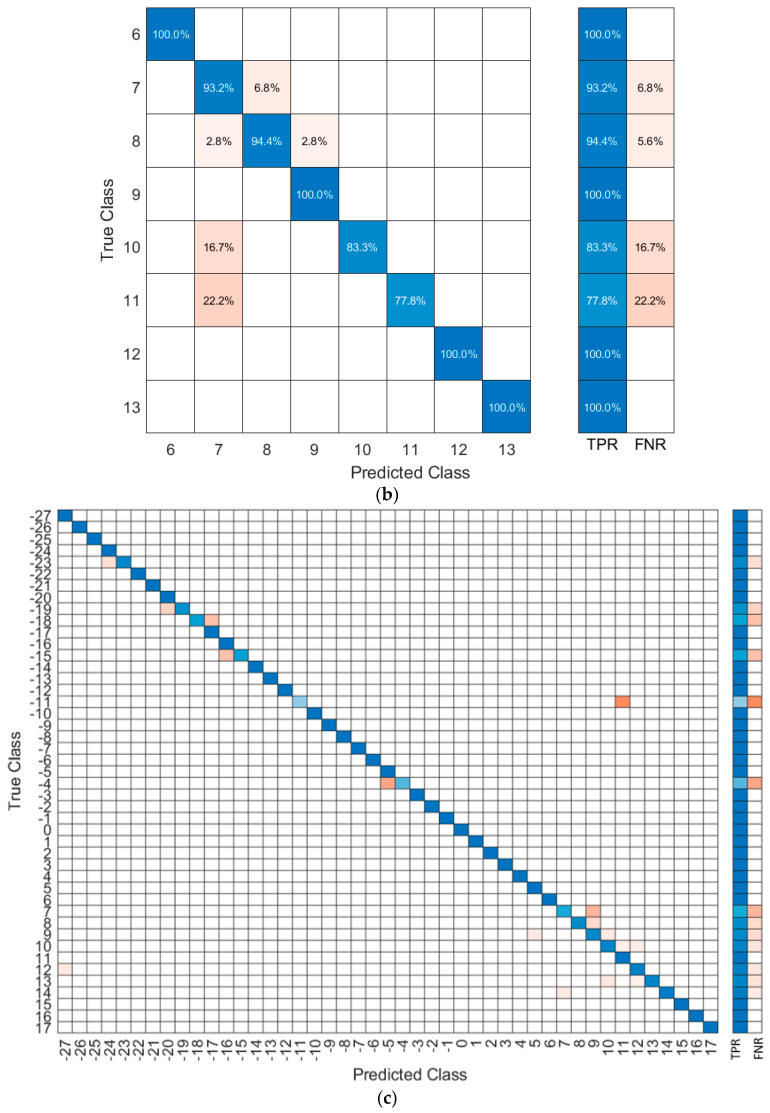

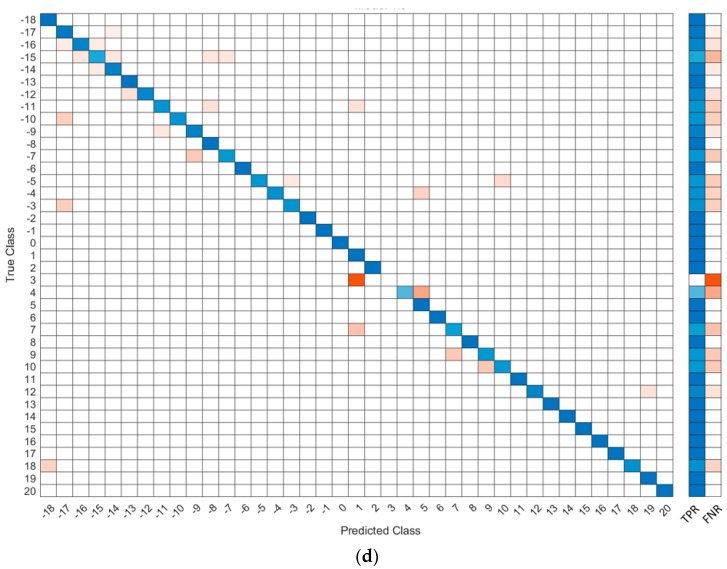
Confusion matrix results for each movement combined size 8.5 and 10.5; (**a**) squat movement left foot confusion matrix; (**b**) squat movement right foot confusion matrix; (**c**) right stoop left foot movement confusion matrix; (**d**) left stoop right foot movement confusion matrix.

The machine learning code was broken up by movement: each movement was analyzed separately to minimize confusion between the movements. As with the graph, the squat was the most accurate movement when training the ankle angle when coupled with the k-nearest neighbor algorithm, returning an accuracy of 93.8%. This algorithm used the six FSRs as inputs to produce the output of the ankle angle. After the accuracy calculations were completed, a confusion matrix was made to determine how the machine learning accurately predicted the outcome. The true angle was located on the *y*-axis while the predicted angle was displayed on the *x*-axis. A perfect model would have had all predictions along the middle diagonal axis. The confusion matrix-generated movements created by the kNN classification algorithm are shown in Figure 9. Figure 9a contains the confusion matrix for the left stoop movement. Figure 9b shows the confusion matrix for the right stoop movement. Figure 9c displays the matrix for a right stoop on the left foot. Figure 9d shows the confusion matrix for the right foot in a left stoop. In the Figures below, the true positive rate (TPR) is the total percentage of positive predictions on the sample set of data given the angle regarded. The false negative rate (FNR) is the total percentage of the incorrect predictions on the sample set of data.

## 4. Discussion

In summary, a footwear-based pressure measurement system implementing standard FSRs to predict the ankle angle using a machine learning-based algorithm was presented. This sensor system could detect and predict the angle of a participant’s ankle with an average accuracy of 89.5% for the squat and stoop movements examined in this study.

Compared with commercially available products such as Zeblok Smart Shoes [30], this prototype could measure joint angles with the addition of measuring gait patterns. The design could do this with minimal sensors, allowing for a cost-effective design compared with on the market solutions. The placement of the FSR allowed for a better prediction of joint angles using a single point instead of pressure zones. The FSR placement coincided with areas of high pressure that showed the largest differential in pressure during the movements associated with the joint angles. A few limitations of the prototype sensor occurred when the shoe was off the ground. The sensor measured the ground reaction force, limiting its ability to measure an angle when the shoe was not planted on the ground.

In the future, instead of using the categorical surrogate model, a linear regression-based model could be used to predict continuous ankle angles. This would allow an angle prediction of a higher decimal precision, if necessary, for future designs. Compared with the existing methods, our proposed shoe-based ankle angle detection system allowed for a comfortable, natural and unnoticeable experience. Therefore, the footwear-based pressure measurement system has a strong potential for utilization in future applications to predict the angle measurements of the user throughout the day. These angle measurements indicate an increased risk for musculoskeletal injury to be addressed prior to injury occurrence. This recognition will lead to a reduction in musculoskeletal injury in the workplace.

## Figures and Tables

**Table 1:** Participant information.

Subject	Sex	Age	Height	Weight	Shoe Size
1	Female	21	5′3″	120	8.5
2	Female	21	5′4″	185	8.5
3	Female	21	5′7”	130	10.5
4	Female	21	5′7”	135	8.5
6	Male	21	5′11”	180	10.5
7	Female	21	5′9″	170	10.5
8	Female	21	5′8″	125	8.5
9	Female	21	5′4″	165	8.5
10	Male	21	6′1″	170	10.5
11	Female	20	5′7″	140	8.5
12	Male	24	5′10″	185	10.5
13	Male	21	5′11″	170	10.5
14	Female	20	5′7″	170	8.5
15	Female	29	5′3″	145	8.5
16	Male	23	5′10″	175	10.5
17	Male	21	6′1″	150	10.5
18	Female	21	5′4″	150	8.5
19	Female	23	5′5″	155	8.5
20	Male	19	6′1″	135	10.5
21	Male	21	5′8″	160	10.5
23	Female	22	5′8″	150	8.5
24	Male	22	5′11″	145	10.5
25	Female	22	5′4″	165	8.5
26	Female	21	5′10″	135	8.5

**Table 2:** Machine learning ankle angle prediction accuracy.

Movement	Foot	Angle Range	Accuracy
Squat	Left	−10 to −3	93.6%
Squat	Right	6 to 13	93.8%
Right Stoop	Left	−27 to 17	89.5%
Left Stoop	Right	−20 to 20	87.4%
